# Antiviral and Antioxidant Activities of Sulfated Galactomannans from Plants of Caatinga Biome

**DOI:** 10.1155/2015/591214

**Published:** 2015-07-14

**Authors:** Márcia Maria Mendes Marques, Selene Maia de Morais, Ana Raquel Araújo da Silva, Naiara Dutra Barroso, Tadeu Rocha Pontes Filho, Fernanda Montenegro de Carvalho Araújo, Ícaro Gusmão Pinto Vieira, Danielle Malta Lima, Maria Izabel Florindo Guedes

**Affiliations:** ^1^Postgraduate Program of Biotechnology, Ceará State University, Avenue Dr. Silas Munguba 1700, Campus do Itaperi, 60714-903 Fortaleza, CE, Brazil; ^2^Course of Chemistry, Ceará State University, Avenue Dr. Silas Munguba 1700, Campus do Itaperi, 60714-903 Fortaleza, CE, Brazil; ^3^Public Health Central Laboratory of Ceará, Avenue Barão de Sturdat 2405, 60120-002 Fortaleza, CE, Brazil; ^4^Technological Development Park (PADETEC), Federal University of Ceará, Campus do Pici, 60455-970 Fortaleza, CE, Brazil; ^5^Health Sciences Center, University of Fortaleza, Avenue Washington Soares 1321, 60811-905 Fortaleza, CE, Brazil

## Abstract

Dengue represents a serious social and economic public health problem; then trying to contribute to improve its control, the objective of this research was to develop phytoterapics for dengue treatment using natural resources from Caatinga biome. Galactomannans isolated from *Adenanthera pavonina* L., *Caesalpinia ferrea* Mart., and *Dimorphandra gardneriana* Tull were chemically sulfated in order to evaluate the antioxidant, and antiviral activities and the role in the inhibition of virus DENV-2 in Vero cells. A positive correlation between the degree of sulfation, antioxidant and antiviral activities was observed. The sulfated galactomannans showed binding to the virus surface, indicating that they interact with DENV-2. The sulfated galactomannans from *C. ferrea* showed 96% inhibition of replication of DENV-2 followed by *D. gardneriana* (94%) and *A. pavonina* (77%) at 25 *µ*g/mL and all sulfated galactomannans also showed antioxidant activity. This work is the first report of the antioxidant and antiviral effects of sulfated galactomannans against DENV-2. The results are very promising and suggest that these sulfated galactomannans from plants of Caatinga biome act in the early step of viral infection. Thus, sulfated galactomannans may act as an entry inhibitor of DENV-2.

## 1. Introduction

Dengue is considered the most important arthropod-borne viral disease in the world in terms of morbidity and mortality in humans. Dengue represents a serious social and economic public health problem in the 21st century [1]. An estimated 50 million dengue infections and approximately 20,000 deaths occur annually [2]. The infection is caused by the dengue virus (DENV), a member of the family Flaviviridae, genus* Flavivirus* and has four antigenically distinct serotypes, DENV-1, DENV-2, DENV-3, and DENV-4. DENV causes a spectrum of disease in humans, ranging from acute febrile dengue fever (DF) to severe forms [3]. There is still no effective treatment against the virus. Thus, extensive efforts have been made toward the development of vaccines and the discovery of potent therapeutic compounds against DENV. Research for dengue antiviral has focused on the different phases of the viral lifecycle (virus attachment, viral entry, trafficking, translation, or replication). Nevertheless, only few antiviral therapies have been tested and little is known about the effects and mechanisms of the antiviral agents [4].

Many studies have confirmed that the naturally sulfated polysaccharides such as polysaccharides from seaweed [5–8] and glycosaminoglycan [9] are potent inhibitors of dengue virus* in vitro* infection, apparently based on the structural similarities to heparan sulfate (HS), a putative receptor present in mammalian cells membranes. HS was reported as receptor molecule for DENV-2 in Vero cells [10]. The sulfated polysaccharides compete with HS for binding to the virus and thus inhibit the entrance of virus in the cell [11, 12].

The importance of free radical molecular species in the pathogenesis of various viral diseases has been increasingly recognized in recent years. Oxygen radicals such as superoxide (O_2_^−^) and hydroxyl radical (OH) have been implicated as possible pathogenic molecules in viral disease pathogenesis [13]. Chen et al. [14] observed that oxidative stress was detected in the* Aedes aegypti* mosquito infected cells. Despite this, the survival of mosquito cells benefits from the upregulation of genes related to antioxidant defence, such as glutathione S transferase (GST).

Viral Infection diseases constitute a major health problem throughout the world. Controlling these diseases is the subject of constant scientific efforts, due to the resistance to known antiviral agents. One very promising approach is the screening of antiviral products derived from natural sources, especially plants.* Adenanthera pavonina* L.,* Caesalpinia ferrea* Mart., and* Dimorphandra gardneriana* Tull are native plants from Caatinga biome of Northeastern* Brazil* and have been used for the treatment of many diseases:* A. pavonina* has been reported to possess antibacterial [15, 16], antioxidant [17], anthelmintic [18], antihyperlipidemic [19], blood pressure lowering [20], and anti-inflammatory [21] effects. Pharmacological properties of* C. ferrea* include hypoglycemic [22, 23], antimicrobial [24], anticancer [25, 26], anti-inflammatory [27, 28], and analgesic [28].

There is a commercial interest in* D. gardneriana* whose fruits are used for rutin extraction, a flavonoid with several antioxidant [29], antiviral [30], antitumor [31], anti-inflammatory [32], and leishmanicidal and anticholinesterase [33] properties. The seeds of this plant are also sources of galactomannans, storage polysaccharides of higher plants. Previous reports indicate the potential of sulfated galactomannans against dengue and yellow fever virus [34], herpes simplex virus [35–37], and poliovirus [37]. As a by-product of the rutin extraction from fruits, the seed galactomannans can be extracted and sulfated to produce sulfated galactomannans to be tested against dengue virus.

In the present study, galactomannans were extracted not only from the seeds of* Dimorphandra gardneriana* (Tul.) but from two other plants from Caatinga biome* Adenanthera pavonina* (L.) and* Caesalpinia ferrea* (Mart.) to establish a comparison between different galactomannans. These polysaccharides were sulfated with chlorosulfonic acid and then the sulfated galactomannans were evaluated for antiradical activity and for inhibitory potential against DENV-2 in Vero cells. This study tried to develop phytoterapics for dengue treatment using abundant natural resources.

## 2. Materials and Methods

### 2.1. Isolation and Purification of Galactomannans

Galactomannans were isolated from the endosperm of seeds of* D. gardneriana*,* A. pavonina,* and* C. ferrea*. The methodology was reported by Vieira et al. [38]. Initially, approximately 73 g of seed species was boiled in 1 L of distilled water for 6 hours to promote enzyme inactivation and depigmentation. Then the seeds were immersed in water for 24 hours, and thereafter the swelling endosperm was manually separated from the embryo and the seed coat and subsequently lyophilized. To obtain the galactomannans, 10 g of isolated and freeze-dried endosperm was subjected to three consecutive exhaustive aqueous extractions. In each extraction 1 L of distilled water at 80°C (constant temperature) was used for a period of 6 hours until formation of a viscous solution. After this procedure, the aqueous extract was vacuum filtered on nylon sieve. The filtrate, a viscous liquid, was stored at 4°C and the solid retained on the sieve was subjected to two further extractions under the same conditions, until the entire endosperm is completely destroyed, leaving only insoluble fibers. At the end of the three exhaustive extractions with the same starting material, the liquids were stored together, with a total of 3 L, which was vacuum filtered (through celite) and lyophilized.

### 2.2. Sulfation of Galactomannans and Purification of the Derivatives

According to Ono et al. [34] and Gamal-Eldeen et al. [39] the galactomannans (300 mg) were added to pyridine:formamide (50 : 10 v/v) with stirring at 25°C (12 hours) until finely dispersed suspensions were obtained. After the dispersion is completed, the mixture was cooled to 4°C and chlorosulfonic acid (4 mL) was slowly added to the mixtures with stirring over 24 hours at 4°C. The resulting solutions were neutralized with saturated aqueous NaHCO_3_, dialyzed (molecular weight cutoff 8–12 kDa) for 120 h against distilled water and then centrifuged (8.800 rpm, 25 min). Three consecutive sulfating reactions were carried out and the sulfated derivate was collected after lyophilizing.

### 2.3. Determination of Sulfate Content in Galactomannans

The sulphate content of the sample (%) was performed by absorption spectrophotometry in the UV-Visible and the degree of sulfation (DS) was determined by a turbidimetric method using the BaCl_2_-gelatin reagent [40]. The method has as principle, the complexion of barium sulfate to form an insoluble salt. Gelatin is used to keep the salt in suspension or increase the settling time of the formed salt. First 200 mg of gelatin (Trade Oxide) is dissolved in 40 mL of hot water at 60–70°C then stored in the refrigerator for 12 hours. Thus, we obtained a mixture of barium and gelatin (transparent gelatinous fluid). After 12 hours, 200 mg of barium chloride was added to the fluid and it was stored in the refrigerator for 3 hours. The polysaccharide was dissolved in 1 M hydrochloric acid and subsequently hydrolyzed for five hours at 105–110°C. A standard solution of anhydrous sodium sulfate (1 mg/mL) was used. After 20 minutes of standing at room temperature, the absorbance was measured at 360 nm and the assay was done in triplicate.

### 2.4. Infrared Spectroscopy Analysis

Infrared spectra (FTIR) of sulfated galactomannans were recorded from KBr pellets on BOMEM spectrometer, scanned between 4.000 and 500 cm^−1^.

### 2.5. Evaluation of Antioxidant Capacity by Inhibition of Free Radical DPPH

To evaluate the antioxidant activity of sulfated galactomannans, the method reported by da Silva et al. [41], with some modifications, adopting the free radical DPPH (1,1-diphenyl-2-picrylhydrazyl), was used. The diluted samples (1 mL) were mixed with 2 mL of methanol solution containing DPPH radicals for 30 min. Ascorbic acid (vitamin C) was used as positive control. The assay was done in triplicate and the tubes were shaken at the end of the additions by vortexing. The absorbance was measured at 517 nm for each concentration and the free radical scavenging effect in percent was calculated using the following formula: [(*A*_0_ − *A* − *A*_*b*_)/*A*_0_] × 100, where *A*_0_, *A*, and *A*_*b*_ indicate the absorbance of the methanol solution of DPPH, sample with solution of DPPH and sample without DPPH solution, respectively. IC_50_ values calculated denote the concentration of the sample required to decrease the absorbance at 517 nm by 50%. The experiment was performed in triplicate.

### 2.6. Cells Culture

Vero cells were cultured in L-15 medium (Leibovitz-Cultilab, Brazil) supplemented with 10% tryptose phosphate broth, 1% penicillin/streptomycin (Gibco-BRL, USD) (50 U/mL), 1% amphotericin B (Gibco-BRL, USD) (250 *μ*g/mL), and 10% fetal bovine serum (FBS) (Sigma-Aldrich, USA) at 37°C in a 5% CO_2_. For maintenance medium (MM), the FBS concentration was reduced to 2%.* Aedes albopictus* C_6/36_ cells were maintained in l-15 medium supplemented with 10% FBS and incubated at 28°C in a 5% CO_2_.

### 2.7. Dengue Virus Preparation

DENV-2 (strain New Guinea) was propagated in C_6/36_ cells. DENV-Infected C_6/36_ cells were incubated at 28°C for 7 days. After time, culture supernatants containing DENV-2 were collected. Viral suspension was prepared as described previously [34] and stored at −80°C. DENV-2 was titrated by cytopathogenicity and expressed as 50% tissue culture infectious dose (TCID_50_)/mL [42] in Vero cells.

### 2.8. Cytotoxicity Evaluation of Sulfated Galactomannans

The cytotoxicity of the sulfated polysaccharides against Vero cells was evaluated by the MTT (4,5-dimethylthiazol-2-yl)-2,5-diphenyl tetrazolium bromide (Sigma-Aldrich, Germany) method, through the quantization of viable cells [30, 43, 44]. The cells were cultured in 96-well plates (TPP, Trasadingen, Switzerland), at a density of 2 × 10^5^ cells/well. After a 24-hour incubation period, at 37°C in an atmosphere of CO_2_, the culture medium was removed and the cells were washed three times with serum-free L-15, and polysaccharides diluted up to 200 *μ*L/mL in MM were added to the cells. Untreated controls were performed by the addition of 200 *μ*L of MM. The cells were then incubated for 7 days. The medium was then removed and 50 *μ*L of MTT solution (5 mg/mL) was added. The plates were reincubated for 4 h. After that, the MTT solution was removed, 100 *μ*L of DMSO was added to dissolve formazan crystals, and the plates were gently shaken, whereby crystals were completely dissolved. The solubilized product was quantified by spectrophotometry at 492 nm (reference at 620 nm). Results were expressed as % inhibition considering absorbance control cells as 100% viable.

### 2.9. Inhibition of Virus Infection by Sulfated Galactomannans

The antiviral activity of the sulfated polysaccharides was also evaluated by the MTT method according to Silva et al. [30] with modification. Briefly, 100 *μ*L of viral suspension (10^2^ TCID_50_/mL) was mixed on ice with 100 *μ*L of sulfated polysaccharides at the indicated concentration and incubated at 37°C for 1 h. The growth medium of confluent Vero cells, prepared at 96-well plates, was removed and cells were washed three times with serum-free L-15. Immediately, the virus-polysaccharides mixtures were inoculated onto the cells monolayers and incubated at 37°C for 1 h. Cell and viral controls were performed by adding only 200 *μ*L of MM or 200 *μ*L of viral suspension, respectively. After the incubation period, the culture medium was removed and cells were again washed three times with serum-free L-15. The MM was added to the cells. Then the cells were incubated for 7 days. The percentages of viability of the dengue virus-infected cells were assayed by MTT colorimetric method as described above for the cytotoxicity assay and values were calculated as [(*A* − *B*) × 100/(*C* − *B*)], where *A*, *B*, and *C* indicate the absorbance of the polysaccharides, virus, and cell controls, respectively.

To detect the prevention of viral infection by sulfated polysaccharides, the assay was designed as follows: a negative control containing Vero cells, a positive control with DENV-2 infected cells, and test groups containing infected cells treated with sulfated polysaccharides. All groups were observed daily up to 7 days after infection by inverted microscope.

### 2.10. Solid-Phase Virus-Binding Assay

The existence of chemical affinity between DENV-2 for sulfated polysaccharides was evaluated through solid-phase virus-binding assay as described previously by Hidari et al. [8] with minor modifications. Briefly, sulfated polysaccharides in phosphate-buffered saline were immobilized on microtiter plates overnight. After blocking with PBS containing 5% BSA, the plates were incubated for 2 h at 28°C with virus purification (5 *μ*g/well). After washing, the plates were incubated for 1 h at 28°C with human anti-dengue antiserum, followed by HRP-conjugated goat anti-human immunoglobulin. The complexes were detected by incubation with o-Phenylenediamine. The absorbance was measured at 492 nm.

### 2.11. Statistical Analysis

All the experiments on the antioxidant effect were calculated as means ± standard deviation (SD). The one-way analysis of variance (ANOVA) test used to determine the statistical differences followed Tukey's multiple comparison tests. The criterion for statistical significance was *P* < 0.05.

## 3. Results and Discussion

This work is the first report of the antioxidant and antiviral effects of sulfated galactomannans from* A. pavonina* (SGAP),* D. gardneriana* (SGDG), and* C. ferrea* (SGCF) against DENV-2 in Vero cells. Ono et al. [34] demonstrated the inhibitory action of sulfated galactomannans from seeds of* Mimosa scabrella* and* Leucaena leucocephala* against DENV-1 in mosquito cells. Sulfated polysaccharides are potent inhibitors of DENV-2 in mammalian cell [5–8, 45, 46]. A differential susceptibility of DENV serotypes to sulfated polysaccharides was showed in Vero and BHK-21 cells, in the order DENV-2 > DENV-3 > DENV-4 > DENV-1 [10].

**Figure 1:**
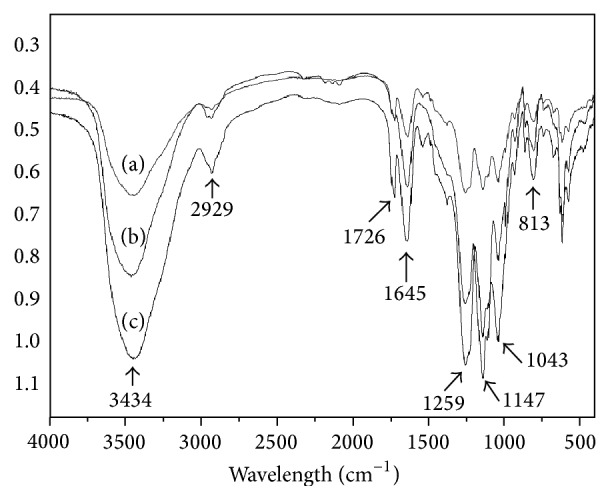
FT-IR of sulfated galactomannans from (a)* Adenanthera pavonina, *(b)* Dimorphandra gardneriana,* and (c)* Caesalpinia ferrea.* The absorption bands at 1259 cm^−1^ correspond to stretching vibrations of the S=O bond.

The presence of sulfate groups in galactomannans derivatives was confirmed by their IR spectra which contained a high-intensity absorption band at 1.259 ± 3 cm^−1^ which was assigned to stretching vibrations of the S=O bond and a moderate intensity band at 813 cm^−1^ indicating a C–O–S vibration (Figure 1). These results indicated that galactomannans from* D. gardneriana, A. pavonina,* and* C. ferrea* seeds were successfully sulfated [47].

To measure Vero cell viability treated with SGAP, SGDG, and SGCF, MTT spectrophometric assay was used. No cytotoxicity was observed for any of the sulfated galactomannans in concentrations of 25, 50, and 100 *μ*g/mL up to 7 days. In all the antiviral experiments 25 *μ*g/mL of each compound was used.

**Figure 2:**
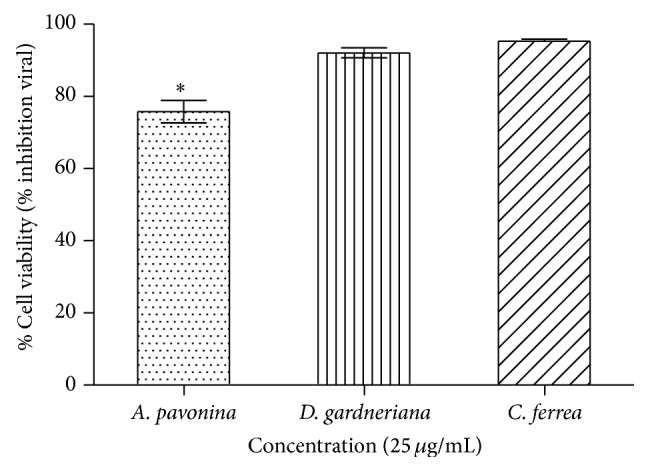
Antiviral activity of sulfated galactomannans: cell viability of the DENV-2-infected Vero cells treated with galactomannans. Statistical significance was determined by Tukey's multiple comparison tests (^*∗*^ *P* < 0.05).

**Figure 3:**
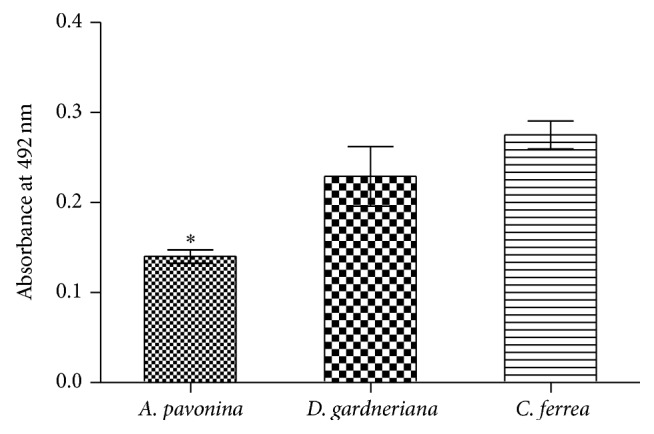
Binding activity of DENV-2 in relation to sulfated galactomannans from* A. pavonina, D. gardneriana,* and* C. ferrea* inimmobilized plastic plates. The bound of dengue virus to compounds was detected by measuring the absorbance at 492 nm. Statistical significance was determined by Tukey's multiple comparison tests (*P* < 0.05).

The use of entrance inhibitor in enveloped viruses is a very attractive strategy for therapeutic intervention, as the site of action of the inhibitor is likely to be extracellular and therefore relatively accessible, and this could also limit cell toxicity [48]. Studies demonstrated that fucoidan and chondroitin sulphate E has antiviral activity against DENV by direct binding of these compounds to the virus [8, 9]. This study showed that DENV-2 bound to SGAP, SGDG, and SGCF (Figure 2), and this binding may be responsible for the inhibitory activity of sulfated galactomannans (Figure 3).

**Figure 4:**
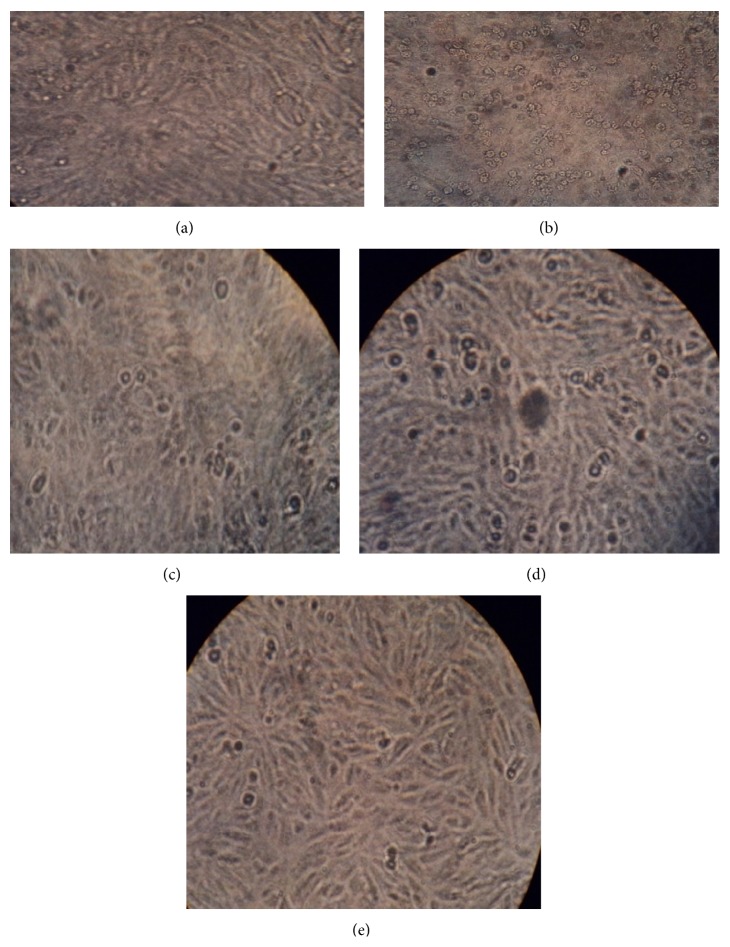
Morphological profile of Vero cell treated or not with sulfated galactomannans (25 *µ*g/mL) up to 7 days after infection (400x). (a) Normal Vero cell (negative control), (b) infected Vero cell with DENV-2, (c) cell treated with* A. pavonina* (SGAP), (d) cell treated with* D. gardneriana* (SGDG), and (e) cell treated with* C. ferrea *(SGCF). The compounds showed protective activity against DENV-2.

The inhibitory potential of SGAP, SGDG, and SGCF against DENV-2 was measured using MTT assay. It was noted that absorbance at 492 nm is directly proportional to the number of living cells in the culture and converted into percent viability, which represent the potency of inhibition by compounds. The measurement of cell viability with values of 70% or above is considered to be prominent with relevant significance [4]. The results demonstrated that all sulfated polysaccharides had inhibitory activity at 25 *μ*g/mL. SGCF exhibited 96% of inhibition against DENV-2, followed by SGDG (94%) e SGAP (77%) (Figure 3). The morphology of cells treated with SGAP, SGDG, and SGCF was shown to be similar to negative control (Vero cells) up to 7 days after infection. As a positive control Vero cells infected with DENV-2 were used; in those, it was observed that about 80% of the monolayer was damaged up to 7 days after infection. Therefore, the results indicated protective activity of sulfated galactomannans (Figure 4).

According to the results of the experiment, it was possible to verify a positive correlation between antiviral activity against DENV-2 and DS (*R* ^2^ = 0.812), and these data corroborate Qiu et al. [49] who mention DS as a relevant parameter for the antiviral activity of a polymer on dengue virus infection. Research has shown that sulfated groups are fundamental for viral action against dengue virus [50]. The sulfated polysaccharides acted as HS-mimetic substances, interfering with the interaction of E glycoprotein with the cellular HS receptor and blocking the interaction.

Ichiyama et al. [51] demonstrated that curdlan sulfate, a sulfated 1→3-*β*-D glucan, has already been tested in humans as inhibition of HIV virus entry without serious side effects and also showed action against dengue virus* in vitro*, which characterizes it as a possible candidate for clinical application. This result opens perspectives to test the sulfated galactomannans SGAP, SGDG, and SGCF in clinical trials, since they displayed inhibitory action against DENV-2 virus without any toxicity in the tested concentrations.

Inhibition of DPPH radical by sulfated galactomannans and vitamin C is shown in Table 1. Wang et al. [47] showed that sulfated galactomannans from guar gum with several DS, in the DPPH test, were found to have strong scavenging activity. In the present work, the SGCF shows also a strong antioxidant activity with IC_50_ = 0.94 *μ*g/mL, much better compared to SGDG and SGAP with IC_50_ = 7.56 and IC_50_ = 7.51 *μ*g/mL, respectively. The sulfated galactomannans from* A. pavonina* (SGAP),* D. gardneriana* (SGDG), and* C. ferrea* (SGCF) had a sulfate content of 33.9%, 35.7%, and 39.0%, respectively. The degree of sulfating (DS) of SGCF (0.82) was higher than that of SGDG (0.75) and SGAP (0.72), as shown in Table 1. The antioxidant activity was related to the DS and results show that sulfate group plays an important role in antiradical activity of sulfated galactomannans. The mechanism of antioxidant action probably is due to hydrogen-donating ability of sulfate groups. According to Teissier et al. [48], sulfated galactomannans from guar gum with several DS, in the DPPH test, were found to have strong antioxidant activities with scavenging property. Viral infections are accompanied with profound changes in cell/tissue metabolism, which lead to generation of reactive oxygen species and may enhance the pathogenesis of the infection. Therefore the use of antioxidants can be of great value in preventing the inception or the progression of the virus disease [52].

## 4. Conclusion

In conclusion, antioxidant and antiviral effects were observed for sulfated galactomannans from seeds of* A. pavonina, D. gardneriana,* and* C. ferrea* and these are directly related to sulfating degree. The investigation of mechanism of action of sulfated galactomannans from plants of Caatinga biome suggests an effect in the early step of viral infection and may act as an entry inhibitor of DENV-2. Further tests are needed to evaluate the antiviral activity of these compounds* in vivo*.

## Figures and Tables

**Table 1:** Sulfate yield (%) of galactomanans and inhibition concentration (IC_50_) of DPPH.

Galactomannans	Sulfate yield (%)	DS	IC_50_ (*μ*g/mL) ± SD
*A. pavonina *	33.9 ± 0.6^a^	0.72 ± 0.04^a^	7.51 ± 0.03^a^
*D. gardneriana *	35.7 ± 0.1^b^	0.75 ± 0.02^a^	7.56 ± 0.04^a^
*C. ferrea *	39.0 ± 0.3^c^	0.82 ± 0.01^b^	0.94 ± 0.01^b^
Vitamin C	—	—	0.48 ± 0.01^b^

Different letters mean significant differences within the lines. IC_50_ means ± standard deviation (SD). DS: sulfating degree.
